# Cellular and Subcellular Compartmentation of the 2*C*-Methyl-D-Erythritol 4-Phosphate Pathway in the Madagascar Periwinkle

**DOI:** 10.3390/plants9040462

**Published:** 2020-04-07

**Authors:** Grégory Guirimand, Anthony Guihur, Catalina Perello, Michael Phillips, Samira Mahroug, Audrey Oudin, Thomas Dugé de Bernonville, Sébastien Besseau, Arnaud Lanoue, Nathalie Giglioli-Guivarc’h, Nicolas Papon, Benoit St-Pierre, Manuel Rodríguez-Concepcíon, Vincent Burlat, Vincent Courdavault

**Affiliations:** 1 Biomolécules et Biotechnologies Végétales, EA 2106, Département of Agronomie, productions animale et végétale et agro-alimentaire, Université de Tours, 31 avenue Monge, 37200 Tours, France; gregory.guirimand@univ-tours.fr (G.G.); anthony.guihur@unil.ch (A.G.); samiramahroug@yahoo.fr (S.M.); audrey.oudin@univ-tours.fr (A.O.); thomas.duge@univ-tours.fr (T.D.d.B.); sebastien.besseau@univ-tours.fr (S.B.); lanoue@univ-tours.fr (A.L.); nathalie.guivarch@univ-tours.fr (N.G.-G.); benoit.stpierre@univ-tours.fr (B.S.-P.); 2 Graduate School of Science, Technology & Innovation, Kobe University, Kobe 657-8501, Japan; 3 Department of Plant Molecular Biology, Faculty of Biology and Medicine, University of Lausanne, 1007 Lausanne, Switzerland; 4 Program of Plant Metabolism and Metabolic Engineering, Centre for Research in Agricultural Genomics (CRAG) CSIC-IRTA-UAB-UB, Campus UAB Bellaterra, 08193 Barcelona, Spain; catalina.perello@gmail.com (C.P.); manuel.rodriguez@cragenomica.es (M.R.-C.); 5 Department of Biology, University of Toronto–Mississauga, Mississauga, 3359 Mississauga Road, ON L5L 1C6, Canada; michaelandrew.phillips@utoronto.ca; 6 Department of Environment Sciences, University of Sidi-Bel-Abbes, 22000 Sidi Bel Abbès, Algeria; 7 Groupe d’Etude des Interactions Hôte-Pathogène (GEIHP, EA 3142), SFR ICAT 4208, Université d’Angers, UNIV. Brest, F-49333 Angers, France; nicolas.papon@univ-angers.fr; 8 Laboratoire de Recherche en Sciences Végétales, Université de Toulouse, CNRS, UPS, 24 chemin de Borde Rouge, Auzeville, BP42617, 31326 Castanet Tolosan, France; burlat@lrsv.ups-tlse.fr

**Keywords:** 2*C*-methyl-D-erythritol 4-phosphate pathway, compartmentation, *Catharanthus roseus*, stromules, GFP imaging, localization artifact, alkaloids

## Abstract

The Madagascar periwinkle (*Catharanthus roseus*) synthesizes the highly valuable monoterpene indole alkaloids (MIAs) through a long metabolic route initiated by the 2*C*-methyl-D-erythritol 4-phosphate (MEP) pathway. In leaves, a complex compartmentation of the MIA biosynthetic pathway occurs at both the cellular and subcellular levels, notably for some gene products of the MEP pathway. To get a complete overview of the pathway organization, we cloned four genes encoding missing enzymes involved in the MEP pathway before conducting a systematic analysis of transcript distribution and protein subcellular localization. RNA in situ hybridization revealed that all MEP pathway genes were coordinately and mainly expressed in internal phloem-associated parenchyma of young leaves, reinforcing the role of this tissue in MIA biosynthesis. At the subcellular level, transient cell transformation and expression of fluorescent protein fusions showed that all MEP pathway enzymes were targeted to plastids. Surprisingly, two isoforms of 1-deoxy-D-xylulose 5-phosphate synthase and 1-deoxy-D-xylulose 5-phosphate reductoisomerase initially exhibited an artifactual aggregated pattern of localization due to high protein accumulation. Immunogold combined with transmission electron microscopy, transient transformations performed with a low amount of transforming DNA and fusion/deletion experiments established that both enzymes were rather diffuse in stroma and stromules of plastids as also observed for the last six enzymes of the pathway. Taken together, these results provide new insights into a potential role of stromules in enhancing MIA precursor exchange with other cell compartments to favor metabolic fluxes towards the MIA biosynthesis.

## 1. Introduction

Isoprenoids, also called terpenoids, constitute a large and highly diverse family of natural products, comprising circa 30,000 distinct compounds in plants. Besides being essential components of the primary metabolism acting as photosynthetic pigments, hormones or redox cofactors, isoprenoids also include specialized metabolites involved in pollinator attraction as well as plant defense against pathogens and herbivores [1,2] Among the defensive terpenoids, plants from the Apocynaceae, Nyssaceae, Loganiaceae, and Rubiaceae families especially synthesize monoterpene indole alkaloids (MIAs) for which more than 2500 distinct structures have been described so far [3,4]. For instance, the Madagascar periwinkle (*Catharanthus roseus*) accumulates more than 130 different MIAs, which limit organ consumption or cause aggressor intoxication upon feeding [5,6,7]. This cytotoxicity also confers highly valuable pharmaceutical properties to several prominent MIAs, such as the antineoplastic vinblastine and vincristine accumulated in *C. roseus*. The high economic value of these two compounds has thus prompted many research groups to elucidate MIA biosynthetic pathways in Apocynaceae, making *C. roseus* a model nonmodel plant [8]. This ultimately led to the characterization of the last steps of vindoline and catharanthine synthesis, the two precursors of vinblastine and vincristine [9,10,11,12,13]. These recent works represent essential advances with a view toward considering the biotechnological production of some interesting MIAs by heterologous hosts [14,15,16].

**Figure 1:**
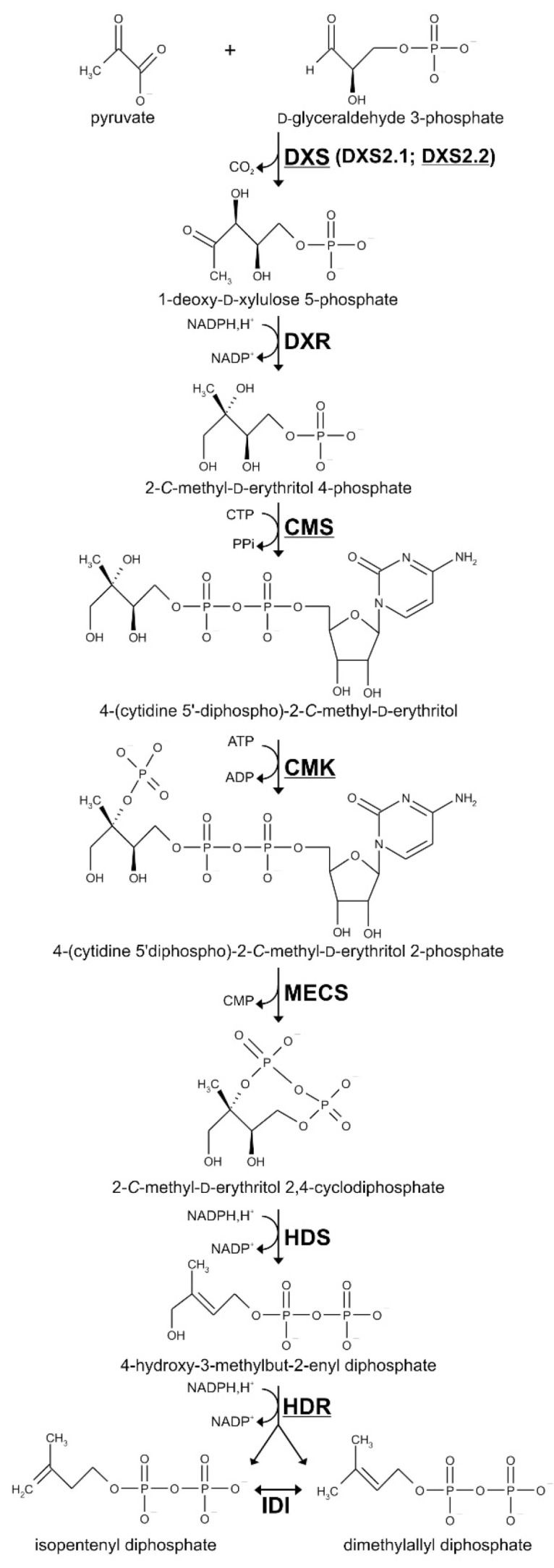
The 2-*C*-methyl-D-erythritol 4-phosphate (MEP) pathway yielding isopentenyl diphosphate (IPP) and its allylic isomer dimethylallyl diphosphate (DMAPP). DXS, 1-deoxy-D-xylulose 5-phosphate synthase; DXR, 1-deoxy-D-xylulose 5-phosphate reductoisomerase; CMS, 2-*C*-methyl-D-erythritol 4-phosphate synthase; CMK, 4-(cytidine 5′diphospho)-2-*C*-methyl-D-erythritol kinase; MECS, 2-*C*-methyl-D-erythritol 2,4-cyclodiphosphate synthase; HDS, (*E*)-4-hydroxy-3-methylbut-2-enyl diphosphate synthase; HDR, (*E*)-4-hydroxy-3-methylbut-2-enyl diphosphate reductase; IDI, isopentenyl diphosphate isomerase.

MIAs exhibit a complex biosynthetic pathway with almost all MIA being derived from strictosidine, which is considered as the first MIA [17]. While multiple decorations and cyclizations of strictosidine yield the plethora of MIAs, its synthesis always starts with the condensation of the indole precursor tryptamine with the monoterpene secoiridoid precursor secologanin. Tryptamine is a shikimate-derived product generated through a single enzymatic step of tryptophan decarboxylation [18]. Secologanin biosynthesis is a more intricate process relying on a nine-step conversion of geranyl diphosphate (GPP), named monoterpene seco-iridoid (MSI) pathway and involving distinct enzyme isoforms [19,20]. Interestingly, the GPP pool engaged in MIA synthesis exclusively comes from the 2*C*-methyl-D-erythritol 4-phosphate (MEP) pathway [21]. The MEP pathway produces both isopentenyl diphosphate (IPP) and dimethylallyl diphosphate (DMAPP) through seven enzymatic reactions initiated by the synthesis of 1-deoxy-D-xylulose 5-phosphate (DXP) from pyruvate and glyceraldehyde 3-phosphate (Figure 1). This reaction is catalyzed by DXP synthase (DXS), encoded by a small gene family in higher plants [22,23,24]. The DXS isogenes have been clustered into two related gene groups: Clade I-DXS including housekeeping genes [25] and Clade II-DXS including genes associated with plant defense and secondary metabolism [23,26]. DXP is then sequentially converted into MEP by DXP reductoisomerase (DXR) and into 4-(cytidine 5′diphospho)-2-C-methyl-D-erythritol (CDP-ME) following the addition of cytidine triphosphate by CDP-ME synthase (CMS).

DXS2.1 and DXS2.2 correspond to class II DXS isoforms. cDNA characterized in this work are underlined. This metabolic intermediate is phosphorylated by CDP-ME kinase (CMK) to form CDP-ME 2-phosphate and cyclized after the loss of the cytidyl group, yielding 2-C-methyl-D-erythritol 2,4-cyclodiphosphate (ME-cPP) in a reaction catalyzed by ME-cPP synthase (MECS). The penultimate step of the MEP pathway relies on the synthesis of (E)-4-hydroxy-3-methylbut-2-enyl diphosphate (HMBPP) catalyzed by HMBPP synthase (HDS) while the final step, catalyzed by HMBPP reductase (HDR), involves the reduction of HMBPP to give a 6:1 mixture of IPP and DMAPP [27]. This unbalanced ratio implicates an additional reaction of IPP isomerization catalyzed by type I IPP isomerase (IDI) to optimize the relative amount of the precursors and to fulfill an efficient biosynthesis of isoprenoids [28]. IPP and DMAPP are then condensed to form GPP that is converted into geraniol to initiate the MSI pathway [29].

In *C. roseus*, the MIA biosynthetic pathway also exhibits complex compartmentation involving no less than four different cell types and eight distinct subcellular compartments [30,31]. Interestingly, all the MEP pathway genes characterized so far, up to the antepenultimate step of the MSI pathway, display a cellular coexpression restricted to cells of the internal phloem associated parenchyma (IPAP) of leaves [19,29,32,33,34,35]. This specific localization thus involves the translocation of the resulting biosynthetic intermediate towards leaf epidermis that houses the subsequent reactions of the pathway. Such a process may represent a regulatory mechanism and a limiting step in the control of the metabolic flux towards MIAs [30]. At the subcellular level, while plastid localization of many MEP pathway enzymes has been described in numerous plants, a plastid targeting has been reported for only HDS and IDI from *C. roseus* [34,35,36]. Interestingly, in addition to plastid stroma localization, these studies also pointed out the localization of both enzymes to stromules. These long protrusions budding from plastids are in close proximity with other organelles such as the endoplasmic reticulum (ER), increasing the putative exchange surface and thus may facilitate transportation of molecules from the main plastid body to other organelles including the ER [37,38]. Additionally, IDI is also targeted to mitochondria and peroxisomes, highlighting the triple targeting of this enzyme [34]. Importantly, while many corroborative data have been gathered over the years, this view of the MEP pathway organization in *C. roseus* remains largely incomplete as less than half of the corresponding genes and proteins has been characterized to date. This prompted us to achieve the cloning and characterization of the missing MEP genes to describe the overall compartmentation of this pathway in *C. roseus*. We thus first focused on the cloning and the functional characterization of genes encoding CMS, CMK, HDR and possible DXS isoforms. Then, we provided a comprehensive overview of both cellular and subcellular distributions of all MEP pathway gene products except IDI that has been thoroughly characterized using the same sample sets in a previous work [34].

## 2. Results

### 2.1. Cloning and Functional Validation of cDNAs Encoding a Second DXS Isoform, CMS, CMK and HDR

Cloning of cDNAs encoding the missing MEP pathway genes (CMS, CMK, HDR) was initiated in the late 2000s before the massive release of *C. roseus* transcriptomic data. Partial cDNAs encoding each enzyme were thus amplified from a *C. roseus* cDNA mixture using degenerate primers. The corresponding full-length coding sequences were subsequently isolated from an orientated *C. roseus* cell cDNA library with specific primers. A similar approach was also used to isolate the second isoform of DXS and phylogenetic studies revealed that both isoforms belong to clade II DXS and were thus named DXS2.1 (initially named DXS) and DXS2.2 (Figure S1; [22,24]). Analysis of the neopublished *C. roseus* transcriptomic data confirmed the existence of all the cloned coding sequences. The deduced amino acid sequences of *C. roseus*, CMS, CMK, HDR and DXS2.2, display a high degree of identity with the corresponding *A. thaliana* orthologs as shown in Table 1. In addition, DXS2.2 exhibits 69% amino acid identity with the previously cloned DXS/DXS2.1. Putative transit peptides (TP) were identified at the N-terminal end of newly characterized enzymes as well as in previously identified ones (Table 1). Depending on the software used for the predictions, either plastid- or mitochondrion-TP were predicted, even though TargetP predictions were always in favor of a plastid targeting.

**Figure 2:**
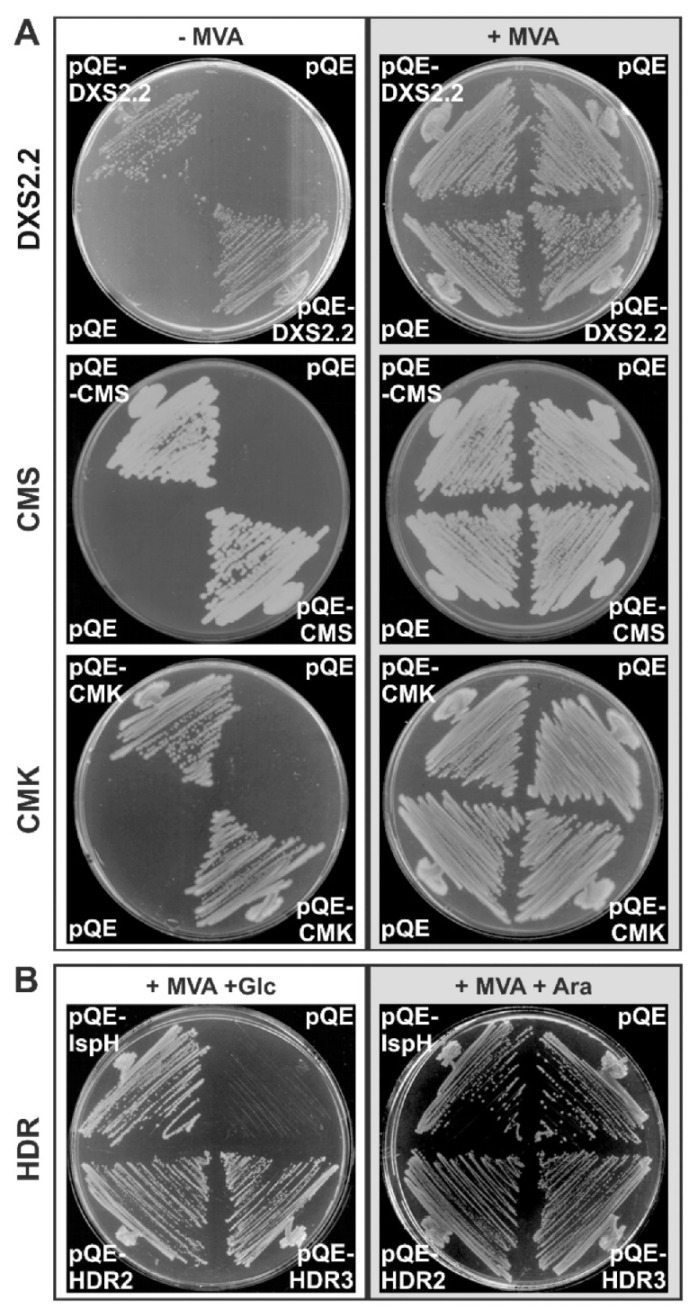
Functional characterization of DXS2.2, CMS, CMK and HDR by complementation of *E. coli* mutant strains. DXS, CMS, CMK-deficient *E. coli* cells (**A**) or HDR-deficient *E. coli* cells (**B**) were transformed with constructs expressing the TP-deleted DXS2.2, CMS, CMK or HDR (HDR2 and HDR3 represent two different deletions of the transit peptide), respectively, or with the original pQE30 vector as a control. After recovering the transformed cells on plates supplemented with 1 mM mevalonate (MVA) to rescue the lethal deletion, colonies were replicated on new plates with (+MVA) or without MVA (−MVA) at 37 °C for DXS, CMS, CMK (**A**) or on plates containing MVA with Glucose (+Glc) or Arabinose (+Ara) for HDR.

Functional validations of DXS2.2, CMS, CMK and HDR were achieved by performing complementation assays in mutant *E. coli* strains, in which a disruption of the corresponding gene results in a lethal phenotype [42,43]. TP-truncated versions of the four proteins were thus expressed in their respective *E. coli* mutant strains using the pQE-30 plasmid. We found that each TP-truncated protein was able to complement the corresponding deficiency of *E. coli* mutants, therefore validating the biochemical activity of the cloned *C. roseus* DXS2.2, CMS, CMK and HDR enzymes (Figure 2A,B). The coding sequences of each enzyme were deposited to Genbank (Table 1).

### 2.2. MEP Pathway Genes Are Highly Coexpressed in IPAP Cells of Young Leaves

**Figure 3:**
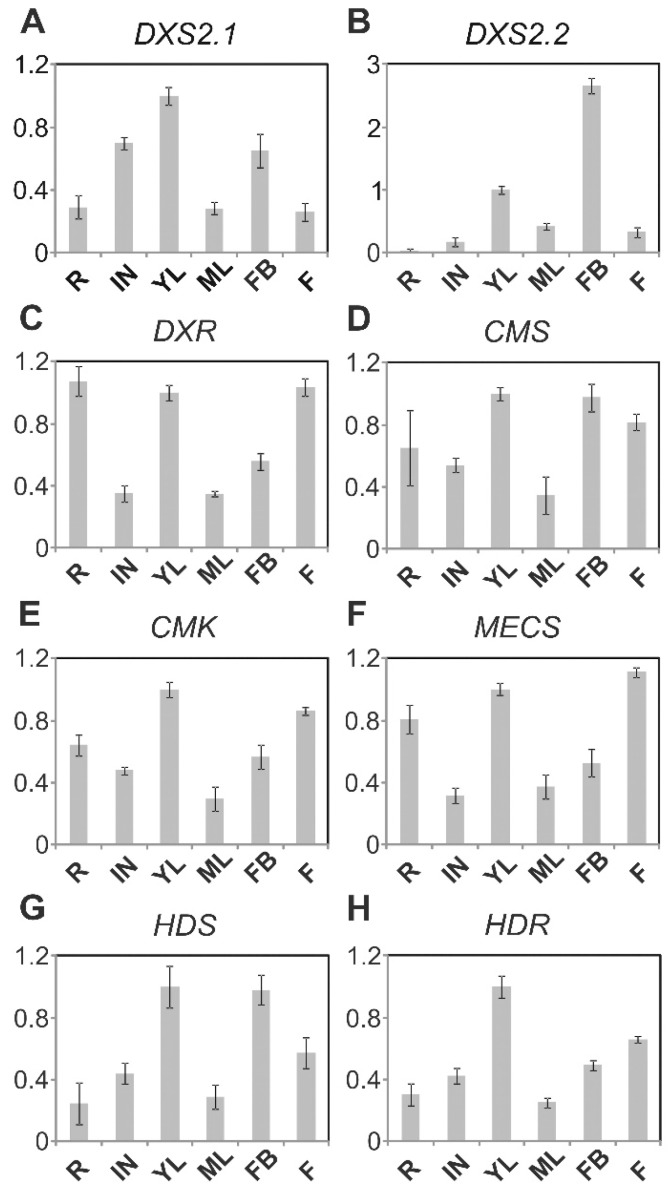
Expressing pattern of MEP pathway genes in different *C. roseus* organs. Total RNA was extracted from the root (R), young leaves (YL), mature leaves (ML), first internodes (IN), flower buds (FB) and mature flower (F), and subjected to reverse transcription. Transcript levels for MEP pathway genes and RPS9 were determined by real-time PCR using gene-specific primers. MEP pathway gene expression levels were normalized using *RPS9*. The transcript level of young leaves was individually set to “1” for the eight genes. (**A**) DXS2.1; (**B**) DXS2.2; (**C**) DXR; (**D**) CMS; (**E**) CMK; (**F**) MECS; (**G**) HDS; (**H**) HDR.

The expression of MEP pathway genes was first analyzed using publically available RNA seq data (Table 2). Interestingly, the coexpression network built using DXS2.1 as a bait illustrated the strong coexpression of all available MEP pathway genes. Six of them were found within the first 20 positions of the network with the Pearson correlation coefficient >0.95. DXS2.2 was ranked 64 with a PCC > 0.90 and HDR was not found on the data set. We further analyzed the expression pattern of the eight genes on fully developed *C. roseus* plants by RT–qPCR. Transcripts of each enzyme were detectable in all tested organs, including young and mature leaves, internodes, flower buds and flowers (Figure 3A–H).

An overall coordinated expression pattern of the whole set of genes, except *DXS2.2*, could be observed with a high accumulation of transcripts in young and actively growing aerial organs (young leaves, flowers buds) but also to a lesser extent in roots and flowers, and with lower levels in internode and mature leaves. Such an expression profile also tightly correlates with that of the IDI gene analyzed on the same samples [34] and is in agreement with the high demand of IPP and DMAPP to fulfill the variety of MEP pathway-derived terpenoid metabolisms in these different organs. By contrast, the distribution pattern of *DXS2.2* transcript distribution was somehow different from *DXS2.1* with barely detectable transcripts in roots and highest expression in flower buds, suggesting that both isoforms could be involved in different physiological processes.

**Figure 4:**
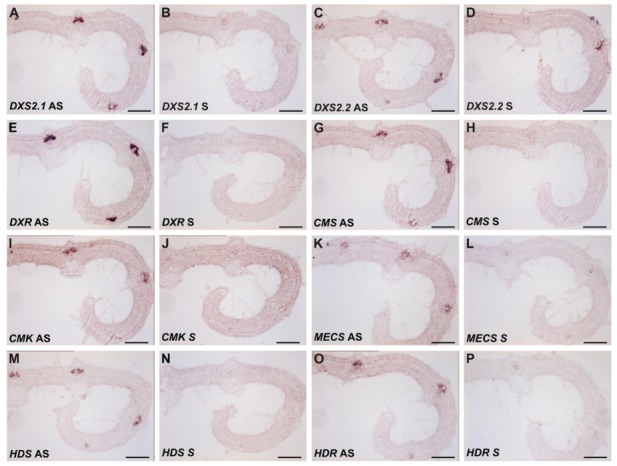
Coexpression of the MEP pathway genes in internal phloem associated parenchyma (IPAP) cells of *C. roseus* young leaves. Paraffin-embedded serial sections of young leaves were hybridized with digoxigenin-labeled transcripts, which were subsequently localized with anti-digoxigenin-alkaline phosphatase conjugates followed by nitro blue tetrazolium chloride (NBT)/5-bromo 4-chloro-3-indolyl phosphate (BCIP) color development. The antisense (AS) probes used for RNA labeling and control hybridization with sense (S) RNA probes are mentioned in the figure. (**A**) DXS2.1 AS; (**B**) DXS2.1 S; (**C**) DXS2.2 AS; (**D**) DXS2.2 S; (**E**) DXR AS; (**F**) DXR S; (**G**) CMS AS; (**H**) CMS S; (**I**) CMK AS; (**J**) CMK S; (**K**) MECS AS; (**L**) MECS S; (**M**) HDS AS; (**N**) HDS S; (**O**) HDR AS; (**P**) HDR S. Bars: 100 µm.

Subsequently, we performed in situ RNA hybridization of *DXS2.2*, *CMS*, *CMK* and *HDR* transcripts on serial sections of young developing leaves, with *DXS2.1*, *DXR*, *MECS* and *HDS* as controls (Figure 4). Using the antisense probes, the transcripts of the four genes were specifically detected in the adaxial part of the vascular region, corresponding to IPAP cells (Figure 4C,G,I,O). Such signal was also observed for *DXS2.1*, *DXR*, *MECS*, *HDS* transcripts as previously established (Figure 4A,E,K,M; [32,35,45,46]) as well as for IDI transcripts analyzed on the same sample [34]. No significant background was observed with the corresponding sense probes (Figure 4B,D,F,H,J,L,N,P). This thus confirms the previous colocalization of *DXS2.1*, *DXR*, *MECS* and *HDS* transcripts and extends it to the herein identified gene transcripts *DXS2.2*, *CMS*, *CMK* and *HDR* transcripts. Taken together, these results thus highlight IPAP as the main site of coexpression of genes of the MEP pathway.

### 2.3. MEP Pathway Enzymes Are Targeted to Plastids

**Figure 5:**
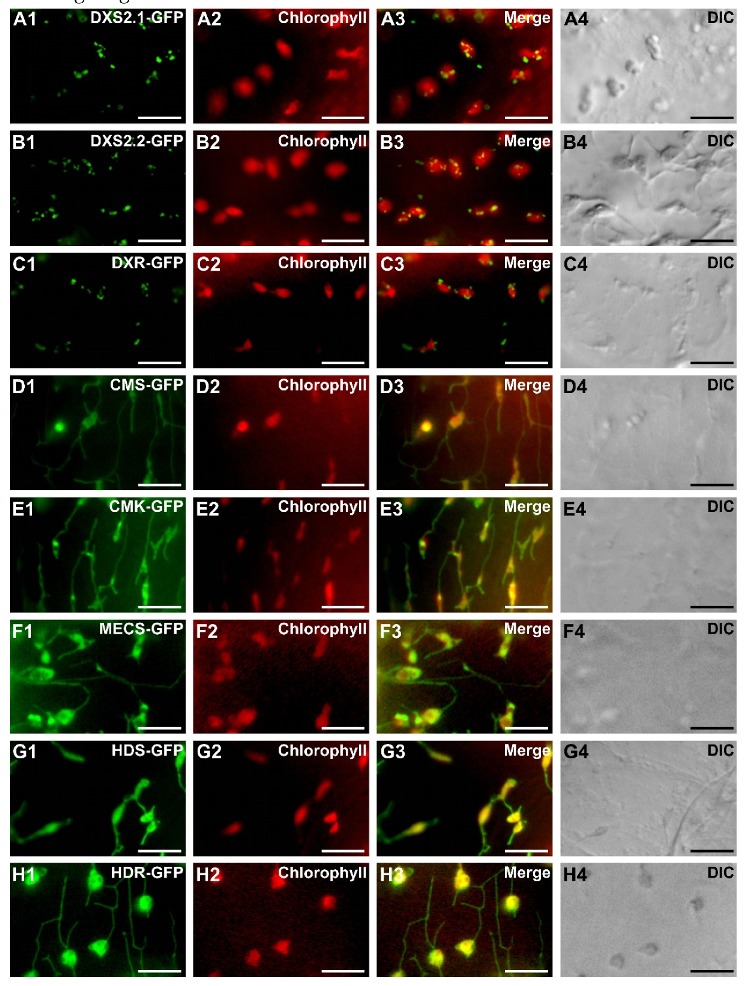
Subcellular localization of the MEP pathway enzymes in *C. roseus* chlorophyll-containing cells. *C. roseus* cells (CR6 cell line) were transiently transformed with plasmids expressing MEP pathway enzymes fused to the N-terminus of GFP as indicated in the first column. The fluorescence emitted by GFP-fused proteins (**A1**–**H1**) was compared to chlorophyll autofluorescence (**A2**–**H2**). Colocalization of the two signals appeared in yellow while merging the two individual (green/red) color images (**A3**–**H3**). Cell morphology was observed with differential interference contrast (DIC, **A4**–**H4**). Bar: 10 µm.

**Figure 6:**
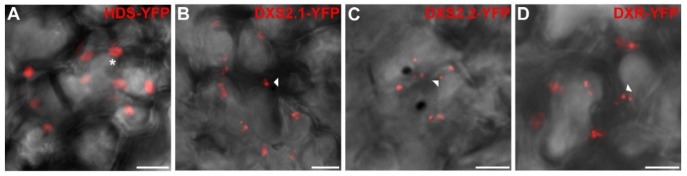
Subcellular localization of HDS-YFP, DXS2.1-YFP, DXS2.2-YFP and DXR-YFP fusions in cells of young *C. roseus* leaf. Young *C. roseus* leaves were transiently transformed with plasmids expressing HDS (**A**) and both DXS (**B**,**C**) and DXR (**D**) fused to the N-terminus of YFP as indicated on the top of each picture. The fluorescence emitted by YFP-fusion proteins was superimposed to cell morphology acquired using differential interference contrast. Arrowhead and star indicate punctuated and diffuse localization in plastids, respectively. Bar: 10 µm.

The subcellular localization of *C. roseus* MEP pathway enzymes was then analyzed through a fluorescent protein (FP) imaging procedure recently developed for *C. roseus* cell cultures [47,48]. The full-length sequence of each enzyme was fused to the N-terminal end of green FP (GFP) to express a MEP enzyme-GFP fusion maintaining accessibility of the predicted TP. The resulting proteins were transiently expressed in *C. roseus* chlorophyll-containing cells for imaging. Depending on enzymes, two different patterns of fluorescence were observed (Figure 5). From CMS to HDR, all enzyme fusions displayed a GFP fluorescence signal colocalizing with the autofluorescence of chlorophylls, demonstrating that these enzymes were targeted to plastids (Figure 5D1–H4). The signal appeared diffused into the stroma, as well as the stromules as previously reported for HDS and IDI [34,36]. In contrast, the two first steps of the MEP pathway, including DXS2.1, DXS2.2 and DXR, displayed a GFP fluorescence signal in punctuated aggregates within plastids (Figure 5A1–C4). Although diffuse fluorescence signals were also observed in plastid stroma of a few numbers of cells, the punctuated aggregates were detected in around 95% of the observed cells for both DXS and DXR. To determine whether this punctuated localization depended on the cell type used for localization, we also performed biolistic-mediated transient transformations of cells from detached *C. roseus* leaves. In these cells, while HDS-Yellow FP (YFP) exhibited a diffuse signal in plastids, both DXS isoforms and DXR YFP fusions still displayed a punctuated fluorescence signal in plastids (Figure 6A–D). Such a protein distribution observed in different cell types was intriguing and raised the question of possible subplastidial targeting of DXS and DXR.

### 2.4. Punctuated Pattern of Localization of DXS and DXR Requires the Whole Protein Sequence

**Figure 7:**
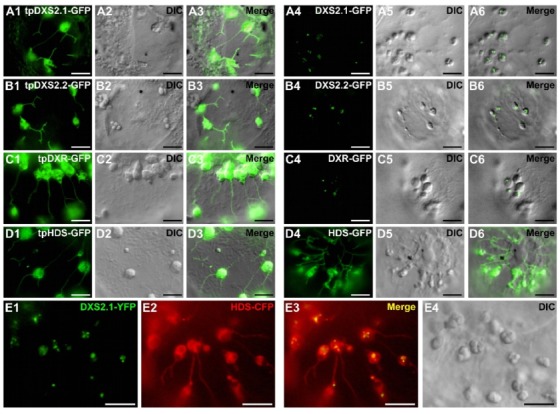
Comparison of the subcellular localization of DXS2.1, DXS2.2, DXR and HDS expressed as full-length-GFP fusion proteins or as transit peptide-GFP fusion proteins. *C. roseus* cells were transiently transformed with plasmids expressing a GFP protein fused to the first 100 residues of the enzyme, encompassing the predicted transit peptide (tp) of DXS2.1 (tpDXS2.1-GFP, **A1**–**A3**), DXS2.2 (tpDXS2.2-GFP, **B1**–**B3**), DXR (tpDXR-GFP, **C1**–**C3**), HDS (tpHDS-GFP, **D1**–**D3**), or fused to full-length DXS2.1 (DXS2.1-GFP, **A4**–**A6**), DXS2.2 (DXS2.2-GFP, **B4**–**B6**), DXR (DXR-GFP, **C4**–**C6**), HDS (HDS-GFP, **D4**–**D6**). An additional co-transformation of DXS2.1-YFP (**E1**) and HDS-CFP (**E2**) was performed to highlight differences of localization in plastids as revealed on the merged image (**E3**). Cell morphology was observed with differential interference contrast (DIC, **E4**). Bar: 10 µm.

**Figure 8:**
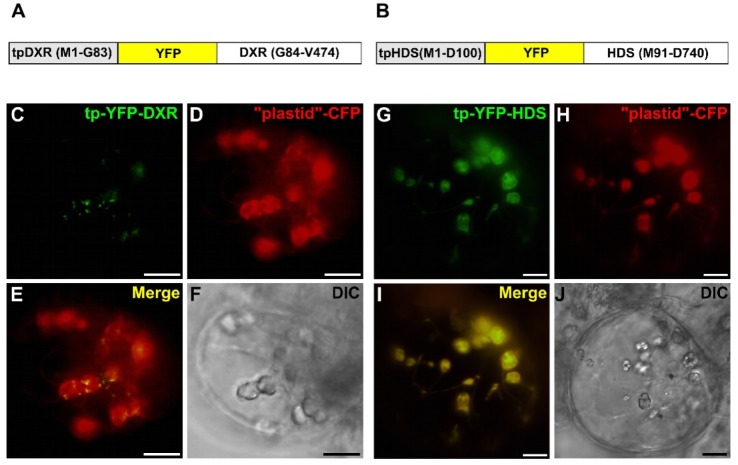
Subcellular localization of DXR and HDS studied using internal YFP protein fusions. *C. roseus* cells were transiently transformed with plasmids expressing internal YFP fused to split DXR (**A**) and HDS (**B**). The fluorescence emitted by YFP-fused proteins (**C**,**G**) was compared to the fluorescence emitted by the plastid-CFP marker (**D**,**H**). Colocalization of the two signals appeared in yellow by merging the two individual (green/red) color images (**E**,**I**). Cell morphology is observed with differential interference contrast (DIC, **F**,**J**). Bar: 10 µm.

To gain insight into the punctuated localization of DXS and DXR, we performed additional fusions with fluorescent proteins. First, to analyze the role of the mature part of these proteins, fusion engaging only the predicted TP (first 100 residues) of DXS2.1, DXS2.2, DXR or HDS (named tpDXS2.1-GFP, tpDXS2.2-GFP, tpDXR-GFP and tpHDS-GFP) were transiently expressed in *C. roseus* cells. Compared to full-length proteins, deletions of the mature part of DXS, DXS2 and DXR led to the loss of the aggregated pattern of fluorescence and resulted in diffuse patterns of fluorescence within the stroma and stromules (Figure 7A1–C6). By contrast, both tpHDS-GFP, HDS-GFP and HDS-CFP displayed a similar diffuse fluorescent signal in the stroma and stromules of plastids (Figure 7D1–D6), clearly distinct from the signal obtained with the DXS2.1-GFP protein (Figure 7E1–E4). In addition, internal fusions with YFP were also created for both DXR and HDS (tpDXR-YFP-DXR and tpHDS-YFP-HDS) (Figure 8A,B). For each of these constructs, the punctuated (DXR) and diffuse plastid (HDS) localizations were conserved (Figure 8C–J), confirming the requirement of the mature part of DXS2.1, DXS2.2 and DXR to their peculiar localization in plastids.

### 2.5. Punctuated Localization of DXS and DXR is Likely Caused by Protein Overexpression

**Figure 9:**
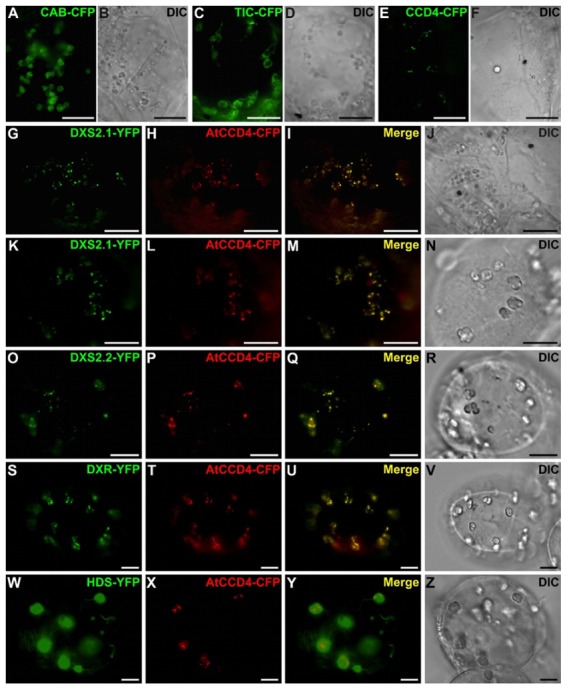
Coexpressions of DXS2.1-YFP, DXS2.2-YFP and DXR-YFP fusions with different plastid markers. Chlorophyll-containing *C. roseus* cells were transiently transformed with plasmids expressing the CFP-marker of thylakoid (CAB-CFP, **A**,**B**), the inner membrane of chloroplast envelope (TIC-CFP, **C**,**D**) and plastoglobuli (Carotenoid Cleavage Dioxygenase (CCD) 4-CFP, **E**,**F**) or cotransformed with plasmids expressing the CCD4-CFP marker and the DXS2.1-YFP fusion protein (**G**–**J**). Chlorophyll-free *C. roseus* cells were transiently transformed with plasmids expressing the CCD4-CFP marker and the DXS2.1-YFP (**K**–**N**), DXS2.2-YFP (**O**–**R**), DXR-YFP (**S**–**V**) and HDS-YFP (**W**–**Z**) fusion proteins. Colocalization of the two signals appeared in yellow by merging the two individual (green/red) color images (**I**,**M**,**Q**,**U**,**Y**). Cell morphology is observed with differential interference contrast (DIC, **B**,**D**,**F**,**J**,**N**,**R**,**V**,**Z**). Bar: 10 µm.

To identify a potential plastid subcompartment hosting DXS2.1-, DXS2.2- and DXR-YFP fusions, we performed cell transformations with distinct markers targeted to thylakoids (CAB-CFP; Figure 9A,B), the inner membrane of plastid envelope (TIC-CFP; Figure 9C,D) and plastoglobuli (CCD4-CFP; Figure 9E,F). While thylakoid and inner membrane markers exhibited circular fluorescent signals in plastids, the plastoglobuli marker displayed a punctuated signal similar to that of DXS2.1, DXS2.2 and DXR fusion proteins. However, cotransformations of cells with plasmids expressing this marker (CCD4-CFP) or DXS2.1, DXS2.2, DXR, HDS-YFP did not reveal a perfect superimposition of the two fluorescent signals, preventing us to firmly conclude on a potential targeting of MEP pathway enzyme to plastoglobuli (Figure 9G–Z).

**Figure 10:**
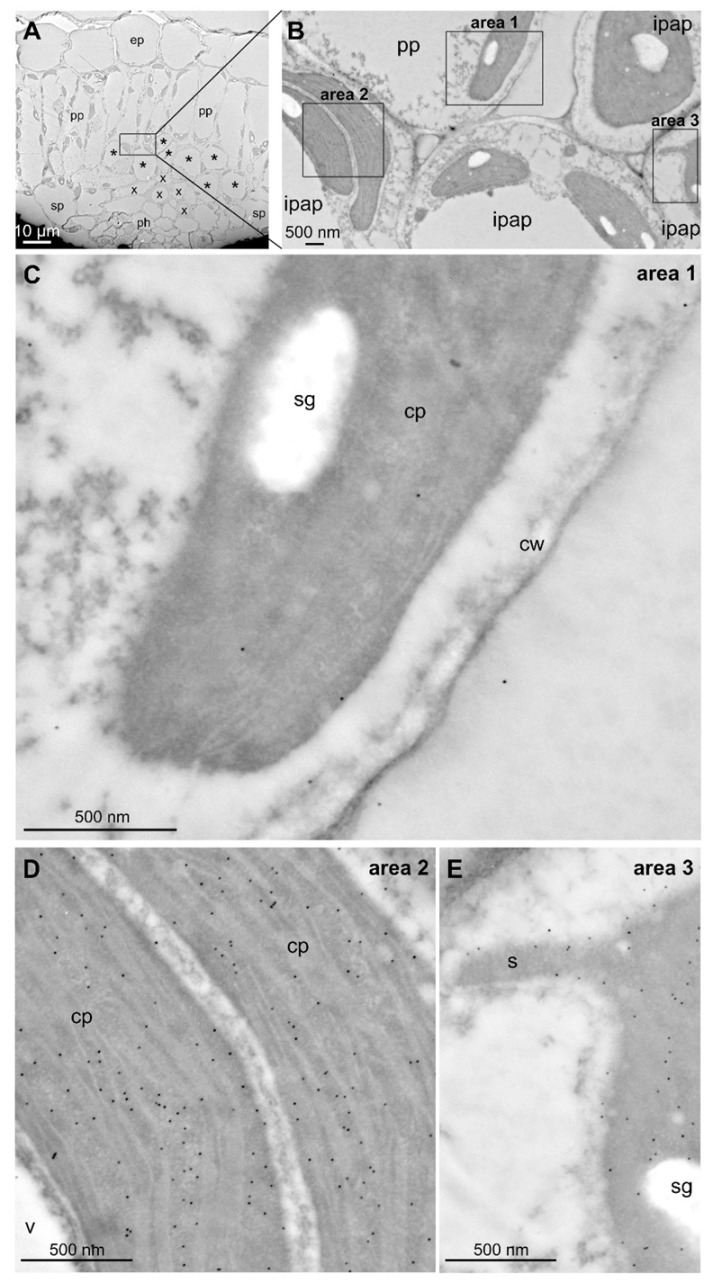
Immunogold labeling of DXR in the stroma of plastids from *C. roseus* leaf cells. (**A**) Morphology of *C. roseus* young leaves observed on ultrathin cross-sections highlighting epidermis (ep), palissadic parenchyma (pp), spongy parenchyma (sp), xylem (x), phloem (ph) and internal phloem associated parenchyma (* or ipap). (**B**) Intermediate magnification highlighting plastids from pp and ipap. (**C**) A very low abundant—but specific—labeling was observed in the stroma of palissadic parenchyma plastids neighboring ipap cells. (**D**,**E**) Typical dense labeling was specifically observed in the stroma of ipap plastids devoid of plastoglobuli-like structures but including stromule budding (s). cp, chloroplast; cw, cell wall; sg, starch granule.

This result prompted us to analyze subcellular localization in native conditions by performing anti-DXR immunogold labeling on cells of *C. roseus* young leaves and transmission electron microscopy (TEM) observation. First, we noticed that the immunogold labeling was only barely detectable in plastids of palissadic parenchyma while a pronounced signal was observed in plastids of IPAP cells (Figure 10A–D), thus confirming our previous results of RNA in situ hybridization (Figure 4).

**Figure 11:**
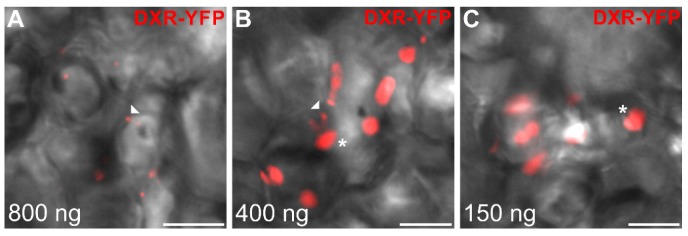
Transient transformation of *C. roseus* young leaf using a decreasing amount of the DXR-YFP expressing construct. Young *C. roseus* leaves were transiently transformed with decreasing amounts of the plasmid expressing DXR fused to the N-terminus of YFP as indicated at the bottom of each picture. The fluorescence emitted by YFP-fusions proteins was superimposed to cell morphology acquired using differential interference contrast. Arrowhead and star indicate punctuated and diffuse localization in plastids, respectively. (**A**) 800 ng; (**B**) 400 ng; (**C**) 150 ng. Bar: 10 µm.

Interestingly, a homogenous immunogold labeling was observed in the stroma of all observed plastids that never displayed plastoglobluli-like structures (Figure 10D,E) but could show some stromule budding including labeling (Figure 10E). Such an absence of the aggregated localization observed through the GFP imaging procedure strongly suggested that it might result from the procedure of protein expression in cells and notably the strong gene overexpression caused by the use of the CaMV35S promoter. To test this hypothesis, additional transformations of the construct expressing the DXR-YFP fusion were performed in cells of *C. roseus* young leaves using a decreasing amount of transforming plasmid DNA. Interestingly, while using high amount of transforming DNA (800 ng per transformation) still resulted in the observation of a punctuated pattern of localization (Figure 11A), lowering bombarded DNA (400 and 150 ng) led to the progressive fading of this localization pattern up to a complete diffuse distribution in plastid stroma (Figure 11B,C). These results therefore confirm the localization of DXR observed through immunolabeling and suggest that DXR is mainly diffuse in plastid stroma. Additionally, these results also support the idea that an artifactual DXS/DXR localization could be caused by gene overexpression.

## 3. Discussion

By cloning the missing genes from the MEP pathway in *C. roseus*, we provide a comprehensive overview of the pathway organization in planta, which may potentially impact its involvement in the synthesis of highly valuable MIAs.

As reported for many plants synthesizing specialized metabolites, we first established that at least two isoforms of clade II DXS coexist, namely DXS2.1 and DXS2.2, in the Madagascar periwinkle (Figure S1). Interestingly, these two isoforms display slightly distinct expression profiles in *C. roseus* organs. DXS2.1 exhibits a coordinated expression with other MEP genes, including IDI, to fulfill IPP and DMAPP to both primary and secondary metabolisms (Figure 3; [30,34]). By contrast, DXS2.2 is mainly expressed in flower buds and much less expressed in roots compared to other MEP genes. This could potentially be linked to a broader involvement of this isoform in the leaf MIA metabolism and the dedicated MIA metabolism taking place in reproductive organs as described previously for a specific isoform of tabersonine 16-hydroxylase [49]. In young leaves, RNA in situ hybridization confirmed that internal phloem associated parenchyma (IPAP) is the main site of expression of MEP pathway genes (Figure 4). Albeit we cannot exclude a lower expression in other leaf tissues, as described with the immunolabeling of HDS and confirmed for DXR (Figure 10; [35]), this result definitively confirms that IPAP is a key tissue in both IPP synthesis and MIA precursor production. The latter thus begins with a DXS-catalyzed reaction up to the antepenultimate step of the MSI pathway catalyzed by 7-deoxyloganic acid hydroxylase and class II cytochrome P450 reductase to form loganic acid [19,50], thus encompassing 16 successive enzymatic steps. While transporters allowing import of loganic acid into the epidermis have been identified, transporters ensuring its export from IPAP have yet to be discovered [51]. The identification of such transporters remains of importance since transport is a key element in the control of the whole MIA metabolism.

**Figure 12:**
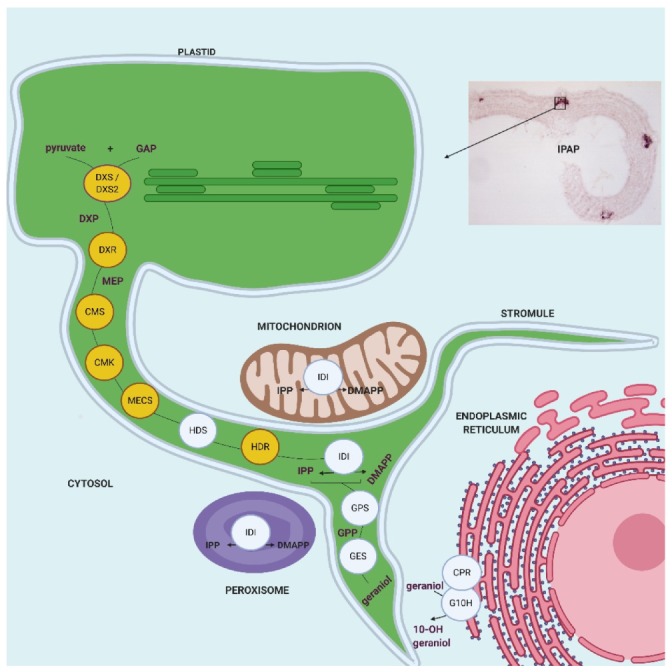
Schematic representation of the cellular and subcellular organization of the MEP pathway and early stage of the monoterpene seco-iridoid (MSI) pathway in *C. roseus* leaves. The enzymes characterized in this study are highlighted in yellow. IDI (type I IPP isomerase) displayed a triple subcellular targeting in stroma/stromules of plastids, mitochondria and peroxisomes as established [34]. The proximity between stromules and the endoplasmic reticulum facilitates export of geraniol in the close environment of G10H to increase hydroxylation rate. DXS, 1-deoxy-D-xylulose 5-phosphate synthase; DXR, 1-deoxy-D-xylulose 5-phosphate reductoisomerase; CMS, 2-*C*-methyl-D-erythritol 4-phosphate synthase; CMK, 4-(cytidine 5′diphospho)-2-*C*-methyl-D-erythritol kinase; MECS, 2-*C*-methyl-D-erythritol 2,4-cyclodiphosphate synthase; HDS, (*E*)-4-hydroxy-3-methylbut-2-enyl diphosphate synthase; HDR, (*E*)-4-hydroxy-3-methylbut-2-enyl diphosphate reductase; IDI, isopentenyl diphosphate isomerase. GPS, geranyl diphosphate synthase. GES, geraniol synthase. CPR, cytochrome P450 reductase. G10H, geraniol 10-hydroxylase. IPAP, internal phloem associated parenchyma. GAP, glyceraldehyde 3-phosphate. DXP, 1-deoxy-D-xylulose 5-phosphate. MEP, 2-C-methyl-D-erythritol 4-phosphate. IPP, isopentenyl diphosphate. DMAPP, dimethylallyl diphosphate. GPP, geranyl diphosphate. Created with Biorender.com.

At the subcellular level, we showed that all MEP pathway enzymes are targeted to plastids including stroma and stromules, as previously established for HDS and IDI [34,35]. A schematic representation of this distribution is given in Figure 12. While DXS2.1, DXS2.2 and DXR were initially reported to display a punctuated pattern of localization in plastids, fusion/deletion experiments, DXR immunolabeling in planta and cell transformations with decreasing amounts of DNA, confirmed that this result was artifactual and probably resulted from artificially high protein expression levels (Figure 5, Figure 7, Figure 9, Figure 10 and Figure 11). These increased amounts of proteins may have impeded the protein folding capacities of DXS and DXR resulting in protein aggregation and formation of the punctuated fluorescence signal. Although partial colocalization with the plastoglobuli markers has been observed (Figure 9), this localization could not be observed with TEM. Additionally, no MEP pathway enzyme has so far been detected in the plastoglobuli proteome, suggesting that early MEP pathway enzymes, while expressed in close proximity to plastoglobuli due to their high density in plastids, are not in fact localized to these structures [52]. Furthermore, such a type of subplastidial distribution has already been described in *Arabidopsis thaliana* for DXS, which is prone to aggregation and is subject to Clp protease-mediated degradation [53,54]. While localization of DXR in extraplastidial vesicles has been reported [53], these vesicles have not been observed in our experimental conditions that mainly rely on transient cell transformations or IPAP TEM observation. These transient transformations are not compatible with the long analysis times required to observe vesicle formation. Alternatively, the development of vesicles for *Arabidopsis* enzymes could be slightly different than for the *C. roseus* counterparts. Based on our results and some previous reports [29,34,55], it appears that plastid stroma and stromules host the first ten steps of the synthesis of the monoterpene precursor of MIAs from DXS up to geraniol synthase. While transporters responsible for export of geraniol into the cytosol remain to be identified, it can be postulated that this subcellular localization particularly involving stromules, may facilitate the release of geraniol in close proximity to the endoplasmic reticulum where geraniol 10-hydroxylase (G10H) catalyzes the subsequent reaction of the MSI pathway [29,30,34,36]. In agreement with this statement, the close proximity between stromules and the endoplasmic reticulum has been already established in *C. roseus* [36] and *Arabidopsis thaliana* [56]. These observations were made by taking advantage of the dynamic properties of GFP imaging procedures while proximity between both organelles are more difficult to characterize through TEM analyses since these rely on cell sectioning and differential organelle electron density (Figure 10). Furthermore, exchanges between stromules and the endoplasmic reticulum have been already characterized, thus supporting geraniol channeling in *C. roseus* [57,58].

In conclusion, besides describing the MEP compartmentation in *C. roseus*, our work also illustrates how high overexpression of proteins may alter their apparent subcellular localization. This should be taken into consideration in the ever-growing number of studies involving this type of technical approach and aiming at manipulating metabolic fluxes in planta.

## 4. Material and Methods

### 4.1. Plant Material and Cell Culture Conditions

Mature *C. roseus* (L) G. Don, cultivar Pacifica Pink (Apocynaceae) plants grown from seeds (Ball Ducrettet, Thonon, France) in a greenhouse were harvested in summer for microscopy fixation (RNA in situ hybridization experiments) and RNA extraction (cloning experiments and gene expression measurements). Young leaves of *C. roseus* (Little Bright Eyes cultivar) grown under similar conditions were used for FP imaging. *C. roseus* cell suspensions used for subcellular localization studies (C20A chlorophyll-free cells and CR6 chlorophyll-containing cells) were propagated in Gamborg B5 medium (Duchefa) at 24 °C under continuous shaking (100 rpm) for 7 days (C20A cells) or 14 days (CR6 cells) as previously described [36].

### 4.2. RNA Extraction and cDNA Synthesis

Plant organs were ground in liquid nitrogen. Total RNA was extracted using the NucleoSpin RNA Plant kit (Macherey-Nagel, France) according to the manufacturer’s instructions. First-strand cDNAs was synthesized from 5 µg of total RNA using 500 ng of oligo(dT)_18_ primers and 15 units of Thermoscript reverse transcriptase (Invitrogen) according to the manufacturer’s instructions. Following retrotranscription, remaining RNA was removed by treatment with *E. coli* RNase H (Invitrogen) during 20 min at 37 °C.

### 4.3. Cloning of the cDNA Sequences from the C. Roseus MEP Pathway Enzymes

The amino acid sequence of CMS, CMK and HDR orthologues from different plant species were retrieved from GenBank and aligned to identify conserved regions. Degenerated primers were designed to amplify an internal cDNA fragment (Table S1). Amplifications were carried out using retrotranscribed RNA extracted from young leaves using an oriented-cells cDNA library as previously described [59]. The corresponding PCR products were purified and cloned into pGEM-T Easy vector (Promega, Charbonnières-les-bains, France) prior to sequencing. A partial cDNA of the second *C. roseus* DXS ortholog was obtained by EST sequencing. Analysis of the five partial cDNA sequences allowed designing specific primers to isolate 5′ and 3′ ends by PCR performed on an oriented *C. roseus* cDNA library using the M13 reverse and T7 universal primers, respectively (Table S1). After sequencing of the resulting PCR products, specific primers were designed to amplify the full-length open reading frame of the five proteins as described for partial cDNA cloning (Table S1). The sequence of the *C. roseus* CMS, CMK, HDR and of the second ortholog of DXS were deposited at NCBI under Genbank accession numbers DQ848672 (DXS2.2), FJ177510 (CMS), DQ848672 (CMK) and DQ848676 (HDR).

### 4.4. Protein Production and E. coli Complementation

Based on ChloroP predictions of plastid transit length (Table 1), truncated cDNA of DXS2.2, CMS, CMK and HDR were amplified as described in Table S2, allowing for removal of the predicted transit peptide and to introduce restriction sites at both cDNA ends. Amplifications were performed using Pfu DNA polymerase (Promega) and the corresponding PCR products were cloned into pQE-30 (Qiagen, Courtaboeuf, France). After sequencing, the plasmids expressing the pseudomature form of DXS2.2, CMS, CMK and HDR were used in bacterial complementation assays as described in Table S2 and according to the procedures described by [42,43].

### 4.5. Gene Expression Measurements (Real-Time PCR Analysis)

The expression of the nine genes of the *C. roseus* MEP pathway and RPS9 gene (GenBank AJ749993) was analyzed by real-time PCR analysis using the specific forward and reverse primers reported in Table S3. Plant organs including roots, first internodes, young and mature leaves, flower buds and flowers, were ground in liquid nitrogen and total RNA was extracted with the RNAeasy Plant mini kit (Qiagen). Total RNA (1.5 µg) was treated with RQ1 RNase-free DNase (Promega) and used for first-strand cDNA synthesis by priming with oligo d(T_17_) (0.5 µM). Reverse transcription was carried out using superscript III RT (Invitrogen) at 50 °C and according to manufacturer’s instructions. Real-time PCR was run on an ABI Prism 7000 SDS light cycler (Applied Biosystems, Foster City, CA, USA) using the SYBR^®^ Green I technology. Each PCR mixture was performed in a total reaction volume of 25 µl containing 1 µl of a 1/3 dilution RT reaction, 0.2 µM forward and reverse primers and 1 × MESA GREEN qPCR master mix Plus buffer (Eurogentec, Angers, France). The reaction was initiated by a denaturation step (95 °C, 10 min), followed by 40 cycles at 95 °C for 15 s and 60 °C for 1 min. RPS9 was used as a housekeeping control gene to allow normalization in each sample. Each assay was performed in triplicate. Data correspond to average values (n = 3) ±standard deviation (SD). Relative transcripts levels in each sample are expressed as a ratio of the abundance of the RPS9 transcripts and normalized to the transcript level of young leaves that was set to one for each gene separately.

### 4.6. Tissue Fixation, Embedding in Paraffin and Microtomy

RNase-free conditions were strictly observed for all steps. Glassware was baked for 8 h at 180 °C and nondisposable plastic ware was incubated 10 min in an aqueous 3% H_2_O_2_ solution and rinsed in DEPC-treated water. Leaves from mature *C. roseus* plants grown in the greenhouse were harvested in late spring/early summer, rapidly fixed in FAA and embedded in Paraplast as previously described [32]. Serial sections (10 µm) were spread on silane-coated slides overnight at 40 °C, and paraffin was removed using xylene (twice for 15 min) before rehydration in an ethanol gradient series up to DEPC-treated water.

### 4.7. RNA In Situ Hybridization

The protocol used for the detection of the MEP pathway transcripts in *C. roseus* leaves was previously described [32,60]. Full-length cDNAs of DXS2.2, CMS, CMK and HDR amplified with the specific corresponding primers (Table S1) and cloned in pSC-A amp/kan (Agilent Technologies) were used for the synthesis of sense and antisense digoxigenin-labeled RNA probes. For DXS2.1, DXR, MECS and HDS, the previously described plasmids were used for the transcription of riboprobes [32,41]. Briefly, RNA hybridization was performed at room temperature, unless otherwise stated. After rehydration, *C. roseus* leaf sections were treated with proteinase K (5 µg·mL^−1^ in 100 mM Tris–HCl and 50 mM EDTA (pH 8.0)) for 30 min at 37 °C, and rinsed twice with TBS150 (10 mM Tris–HCl, 150 mM NaCl (pH 7.5)), followed by blocking of proteinase K with glycine (2 mg·mL^−1^ in TBS150) for 2 min, and by two rinses in TBS150. After postfixation with 3.7% formaldehyde in PBS during 20 min and two washing steps in TBS150 (5 min), sections were acetylated with acetic anhydride (0.25% in 0.1 M triethanolamine–HCl (pH 8.0)) for 10 min, washed with TBS150, dehydrated in an ethanol series and air-dried. To detect RNA, 120 µL of the hybridization mix (200 ng·mL^−1^ partially hydrolyzed digoxigenin-labeled RNA transcripts, 40% formamide, 10% dextran sulfate, 1 µg·mL^−1^ deproteinised yeast total RNA, 0.3 M NaCl, 0.01 M Tris–HCl (pH 6.8), 10 mM sodium phosphate (pH 6.8), 5 mm EDTA and 40 units·mL^−1^ RNasin ribonuclease inhibitor) were dispersed on the sections before mounting under cover slips to limit evaporation. Hybridization was performed during 16 h at 50 °C in an atmosphere containing 50% formamide. Cover slips were removed from slides through soaking in 2 × SSC at 37 °C (1 × SSC is 0.15 M NaCl and 15 mM sodium citrate). Before detection, slides were treated with RNase A (50 µg·mL^−1^ in 10 mM Tris–HCl, 0.5 M NaCl, 1 mM EDTA (pH 7.5)) for 30 min at 37 °C followed by washing under gentle agitation in 2 × SSC for 1 h, 1 × SSC for 1 h and in 0.1 × SSC for 1 h at 65 °C. To detect hybridized transcripts, slides were washed in Tween TBS (TTBS) (0.1 M Tris–HCl (pH 8.0), 0.15 M NaCl and 0.3% Triton X-100) for 10 min and blocked with 2% BSA in TTBS for 30 min. Sheep antidigoxigenin Fab fragments–alkaline phosphatase conjugate (Roche) at a 1:200 dilution in a solution of 1% BSA in TTBS was dispensed on the sections and mounted under cover slips. After incubation for 2 h, unbound conjugates were washed two times for 15 min with TTBS and two times for 10 min with AP buffer (0.1 M Tris–HCl (pH 9.5), 0.1 M NaCl and 10 mM MgCl_2_). For color development, slides were immerged in 175 µg·mL^−1^ 5-bromo-4-chloro-3-indolyl phosphate (BCIP) and 350 µg·mL^−1^ nitro blue tetrazolium chloride in AP buffer for 1–16 h. After development, slides were washed in water, dried and mounted in immersion oil under cover slips.

### 4.8. Bioinformatic Sequence Analysis

The predictions of protein subcellular localization were performed using PSORT [61], Predotar [62], ChloroP 1.1 [63], MitoProt [64] and TargetP 1.1 [65] softwares.

### 4.9. Coexpression Network Analysis

The MPGR RNA seq data [44] were analyzed using Microsoft Excel. DXS2.1 was used as a bait to build a coexpression network with Pearson correlation coefficient for the 23 sample conditions. The network was ranked and filtered for the six other MEP pathway available genes (HDR was not present). A relative red-yellow-green heat map was used to highlight the expression profiles.

### 4.10. FP-Fusion Constructs for MEP Pathway Enzymes Localization Studies

Plasmids expressing GFP and/or YFP fusion proteins of the MEP pathway enzymes including their orientation or truncated variants were constructed using the plasmids and the procedures previously described [36]. Details on primers and on cloning procedures are listed in Table S4 for full-length fusion, in Table S5 for the fusion including the first 100 residues of the DXS2.1, DXS2.2, DXR and HDS and in Table S6 for DXR and HDS internal YFP fusions.

### 4.11. Organelle Markers

For the identification of the subcellular compartments that accumulate the fusion proteins, a set of organelle markers was used in cotransformation experiments with the MEP pathway enzyme constructs. The “plastid”-mcherry (CD3-1000), “plastid”-CFP (CD3-994), “mitochondria”-mcherry (CD3-992), “mitochondria”-CFP (CD3-986), and “peroxisome”-CFP markers, described by [66] were obtained from the ABRC (http://www.arabidopsis.org). The mcherry-GUS cytosolic marker and the mcherry nucleocytosolic marker were described previously [59]. For plastid subcompartment markers, the CAB1 (chlorophyll a/b binding protein 1, At1g29930), TIC40 (translocon at the inner envelope of chloroplasts, At5g16620) and CCD4 (carotenoid cleavage dioxygenase 4, At4g19170) proteins of *A. thaliana* were used as thylakoid, inner membrane of plastid and plastoglobuli markers, respectively. The plastoglobule localization of CCD4 was previously demonstrated by [67] and [52]. The coding sequence of the three proteins was amplified using specific primers (Table S7) and retrotranscribed from RNA extracted from *A. thaliana* as a matrix, and subsequently cloned into the pSCA-cassette-CFPi plasmid [59].

### 4.12. Cell Transformations and Epifluorescence Microscopy

Transient transformation of *C. roseus* cells by particle bombardment, fluorescence protein imaging and BiFC were performed following the procedures previously described [36,47,48]. Briefly, *C. roseus* cells plated onto solid culture medium or ditched young leaves were bombarded with gold DNA coated particles (1 µm) and 1100 psi rupture disk at a stopping-screen-to-target distance of 6 cm, using the Bio-Rad PDS1000/He system. Cells were cultivated during 15 h to 48 h and the protein subcellular localization was determined using an Olympus BX-51 epifluorescence microscope equipped with an Olympus DP-71 digital camera. The morphology of the transformed cells was observed with differential interference contrast (DIC). The pattern of localization presented for each protein is representative of circa 100 observed cells.

### 4.13. Transmission Electron Microscopy Immunogold Labeling

Transmission electron microscopy immunogold labeling was performed according to [41] using the anti-DXR antibody described in [68] at 1:5 to 1:10 dilution. The observations were performed using the electron microscopy facilities of the Toulouse Imaging Network.

## Figures and Tables

**Table 1:** Identity of the *C. roseus* MEP pathway enzymes excluding transit peptides with the corresponding *A. thaliana* orthologs and prediction of subcellular localization of the full sequences.

Enzyme	Identity	PSORT	Predotar	ChloroP	MitoProt	TargetP
DXS/DXS2.1	74% 64% 41%	ER (membrane) 0.550 Peroxisome 0.102 ER (lumen) 0.100	P 0.45 M 0.01 E 0.51	Y (0.509) 35 residues	0.636 37 residues	cTP (0.375) mTP (0.217)
DXS2.2	65% 56% 34%	P (stroma) 0.570 M (matrix) 0.475 Cyto 0.450	P 0.91 M 0.06 E 0.08	Y (0.565) 62 residues	0.327 16 residues	cTP (0.849) mTP (0.301)
DXR	89%	P (stroma) 0.520 Cyto 0.450 M (matrix) 0.360	P 0.30 M 0.01 E 0.69	Y (0.509) 83 residues	0.596 66 residues	cTP (0.303) mTP (0.049)
CMS	76%	P (stroma) 0.865 P (thy mb) 0.515 P (thy space) 0.461	P 0.07 M 0.20 E 0.71	Y (0.576) 66 residues	0.5259 Not predictable	cTP (0.932) mTP (0.083)
CMK	75%	P (stroma) 0.899 P (thy mb) 0.595 P (thy space) 0.595	P 0.84 M 0.01 E 0.16	Y (0.582) 57 residues	0.949 59 residues	cTP (0.895) mTP (0.084)
MECS	85%	Plasma mb 0.700 P (thy mb) 0.547 M (inner mb) 0.415	P 0.97 M 0.03 E 0.03	Y (0.582) 55 residues	0.5829 58 residues	cTP (0.955) mTP (0.024)
HDS	87%	ER (lumen) 0.850 Plasma mb 0.790 Peroxisome 0.300	P 0.66 M 0.02 E 0.33	Y (0.526) 40 residues	0.009 18 residues	cTP (0.496) mTP (0.098)
HDR	79%	M (matrix) 0.850 Peroxisome 0.322 Nucleus 0.300	P 0.80 M 0.02 E 0.20	Y (0.530) 34 residues	0.104 Not predictable	cTP (0.827) mTP (0.129)

DXS/DXS2.1 (CAA09804, [39]); DXS2.2 (ABI35993, this work); DXR (AAF65154, [40]), CMS (ACI16377, this work); CMK (ABI35992, this work); MECS (AAF65155, [40]), HDS (AAO24774, [41]); HDR (ABI30631, this work). For DXS2.1 and DXS2.2, the percent of identity has been established with DXS/CLA1, DXSL2/DXL1, DXS3/DXL2, respectively. For the PSORT and Predotar predictions of subcellular localization, the three most favorable localizations are indicated with the corresponding score. For the ChloroP, MitoProt and TargetP predictions, scores of transit peptide presence and transit peptide length are given. cTP, chloroplast transit peptide; Cyto, cytoplasm; E, elsewhere; ER, endoplasmic reticulum; M, mitochondria; mb, membrane; mTP, mitochondria transit peptide; P, plastid; thy, thylakoid.

**Table 2:** MEP pathway gene RNA seq coexpression pattern. A *DXS2.1* coexpression network was drawn using Medicinal Plant Genomics Ressource MPGR RNA seq data [44] showing the clustering of the MEP pathway genes. The codes for the 23 samples and the transcript accession numbers (i.e., cra locus numbers) are from MPGR. See methods for details.

RANK	1	2	8	14	16	20	64	NF
**PCC**	**1**	**0,98859**	**0,96838**	**0,96244**	**0,96168**	**0,955751**	**0,921181**	**NF**
**CRA LOCUS**	**618**	**4910**	**1424**	**7966**	**891**	**4962**	**381**	**2720**
**NAME**	**DXS2.1**	**CMK**	**HDS**	**MECS**	**CMS**	**DXR**	**DXS2.2**	**HDR**
**CRA_AA**	**23**	**66**	**149**	**40**	**26**	**69**	**9**	**NF**
**CRA_AN**	**49**	**65**	**144**	**82**	**33**	**65**	**15**	
**CRA_AM**	**36**	**81**	**161**	**89**	**30**	**92**	**9**	
**CRA_AO**	**34**	**37**	**48**	**27**	**10**	**52**	**16**	
**CRA_AP**	**221**	**113**	**239**	**87**	**39**	**138**	**45**	
**CRA_AE**	**85**	**70**	**109**	**57**	**23**	**71**	**16**	
**CRA_AG**	**416**	**227**	**287**	**126**	**46**	**216**	**106**	
**CRA_AF**	**191**	**129**	**242**	**96**	**45**	**132**	**100**	
**CRA_AH**	**52**	**32**	**105**	**58**	**10**	**40**	**19**	
**CRA_AB**	**65**	**44**	**164**	**48**	**13**	**42**	**9**	
**CRA_AC**	**26**	**26**	**106**	**41**	**10**	**22**	**5**	
**CRA_AD**	**22**	**25**	**98**	**36**	**12**	**26**	**4**	
**CRA_AL**	**26**	**21**	**78**	**32**	**7**	**26**	**5**	
**CRA_AI**	**38**	**29**	**85**	**42**	**14**	**23**	**15**	
**CRA_AJ**	**41**	**33**	**74**	**41**	**15**	**28**	**19**	
**CRA_AK**	**35**	**34**	**72**	**32**	**5**	**26**	**5**	
**CRA_AS**	**102**	**103**	**206**	**78**	**31**	**173**	**36**	
**CRA_AQ**	**181**	**146**	**310**	**135**	**51**	**254**	**104**	
**CRA_AR**	**67**	**60**	**151**	**75**	**15**	**123**	**30**	
**CRA_AT**	**141**	**110**	**234**	**66**	**29**	**243**	**42**	
**CRA_AU**	**803**	**534**	**1084**	**382**	**146**	**1374**	**256**	
**CRA_AV**	**186**	**148**	**275**	**86**	**41**	**281**	**79**	
**CRA_AW**	**832**	**573**	**1154**	**335**	**136**	**1288**	**529**	

## Supplementary Materials

The following are available online at https://www.mdpi.com/2223-7747/9/4/462/s1, Figure S1: A phylogenetic tree was constructed from the amino acid sequence of DXS from the following species. Table S1: Details of primer sequences and cloning procedure used to amplify the DX2.2, CMS, CMK and HDR cDNAs. Table S2: Details of primer sequences and cloning procedures used to express proteins for functional validation. Table S3: The sequence of primers used in qRT–PCR analysis. Table S4: Details of primer sequences and cloning procedure used to express full-length MEP pathway enzyme fused to FP. Table S5: Details of primer sequences and cloning procedure used to fuse the first 100 residues of DXS2.1, DXS2.2, DXR and HDS to GFP. Table S6: Details of primer sequences and cloning procedures used to create internal YFP fusion for DXR and HDS. Table S7: Details of primer sequences and cloning procedures used to create plastid subcompartment CFP-markers; the CAB1 (chlorophyll a/b binding protein 1, At1g29930), TIC40 (translocon at the inner envelope of chloroplasts, At5g16620), CCD4 (carotenoid cleavage dioxygenase 4, At4g19170) proteins of *A. thaliana* were used as a marker of thylakoid, inner membrane of plastid and plastoglobules, respectively. The coding sequence of these proteins was amplified using retrotranscribed RNA extracted from *A. thaliana* as a matrix.
