# Supplementary material for: Cellular and Subcellular Compartmentation of the 2C-Methyl-D-Erythritol 4-Phosphate Pathway in the Madagascar Periwinkle
Source: Plants (Basel). 2020 Apr 7;9(4):462. doi: 10.3390/plants9040462 (PMC7238098; doi:10.3390/plants9040462)
Supplement: Supplementary file 1 [file plants-09-00462-s001.pdf]

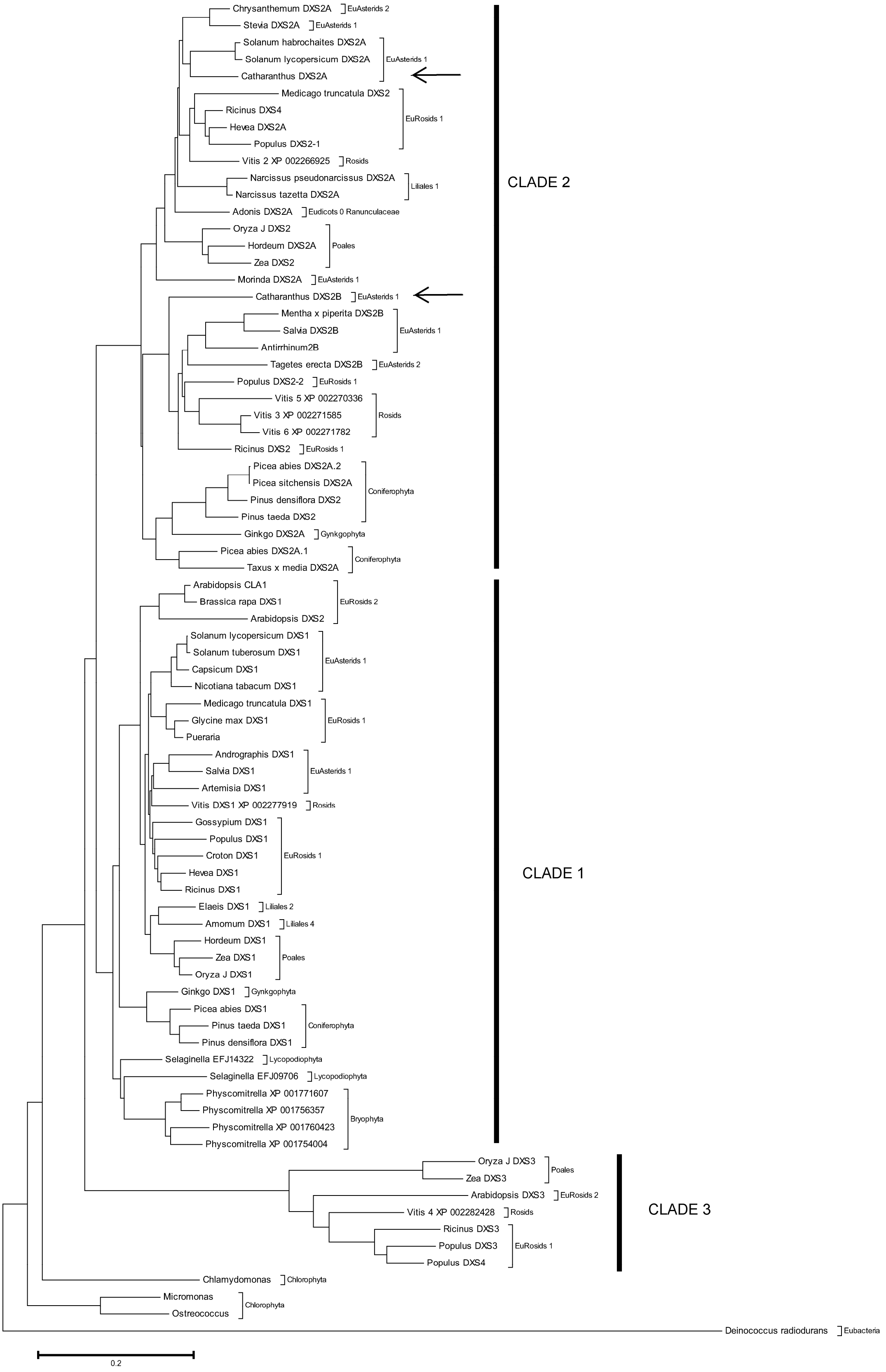
Chrysanthemum DXS2A
EuAsterids 2
Stevia DXS2A
EuAsterids 1
Solanum habrochaites DXS2A
Solanum lycopersicum DXS2A
EuAsterids 1
Catharanthus DXS2A
Medicago truncatula DXS2
Ricinus DXS4
EuRosids 1
Hevea DXS2A
Populus DXS2-1
Vitis 2 XP 002266925
Rosids
Narcissus pseudonarcissus DXS2A
Liliales 1
Narcissus tazetta DXS2A
Adonis DXS2A
Eudicots 0 Ranunculaceae
Oryza J DXS2
Hordeum DXS2A
Poales
Zea DXS2
Morinda DXS2A
EuAsterids 1
Catharanthus DXS2B
EuAsterids 1
Mentha x piperita DXS2B
Salvia DXS2B
EuAsterids 1
Antirrhinum2B
Tagetes erecta DXS2B
EuAsterids 2
Populus DXS2-2
EuRosids 1
Vitis 5 XP 002270336
Vitis 3 XP 002271585
Rosids
Vitis 6 XP 002271782
Ricinus DXS2
EuRosids 1
Picea abies DXS2A.2
Picea sitchensis DXS2A
Coniferophyta
Pinus densiflora DXS2
Pinus taeda DXS2
Ginkgo DXS2A
Gynkgophyta
Picea abies DXS2A.1
Coniferophyta
Taxus x media DXS2A
CLADE 2
Arabidopsis CLA1
Brassica rapa DXS1
EuRosids 2
Arabidopsis DXS2
Solanum lycopersicum DXS1
Solanum tuberosum DXS1
EuAsterids 1
Capsicum DXS1
Nicotiana tabacum DXS1
Medicago truncatula DXS1
Glycine max DXS1
EuRosids 1
Pueraria
Andrographis DXS1
Salvia DXS1
EuAsterids 1
Artemisia DXS1
Vitis DXS1 XP 002277919
Rosids
Gossypium DXS1
Populus DXS1
Croton DXS1
EuRosids 1
Hevea DXS1
Ricinus DXS1
Elaeis DXS1
Liliales 2
Amomum DXS1
Liliales 4
Hordeum DXS1
Zea DXS1
Poales
Oryza J DXS1
Ginkgo DXS1
Gynkgophyta
Picea abies DXS1
Pinus taeda DXS1
Coniferophyta
Pinus densiflora DXS1
Selaginella EFJ14322
Lycopodiophyta
Selaginella EFJ09706
Lycopodiophyta
Physcomitrella XP 001771607
Physcomitrella XP 001756357
Bryophyta
Physcomitrella XP 001760423
Physcomitrella XP 001754004
CLADE 1
Oryza J DXS3
Poales
Zea DXS3
Arabidopsis DXS3
EuRosids 2
Vitis 4 XP 002282428
Rosids
Ricinus DXS3
Populus DXS3
EuRosids 1
Populus DXS4
CLADE 3
Chlamydomonas
Chlorophyta
Micromonas
Chlorophyta
Ostreococcus
Deinococcus radiodurans
Eubacteria
0.2

Supplemental Figure S1. A phylogenetic tree was constructed from the amino acid sequence of DXS from the following species : *Adonis aestivalis* (ABK35283.1) ; *Amomum villosum* (ACR02668.1) ; *Andrographis paniculata* (AAP14353.1) ; *Arabidopsis thaliana* (CLA1 At4g15560 ; DXS2 At3g21500; DXS3 AT5G11380); *Artemisia annua* (AAD56390.2) ; *Antirrhinum majus* (AAW28999.1) ; *Brassica rapa* (ABE60813.1) ; *Capsicum annuum* (CAA75778.1) ; *Catharanthus roseus* (DXS2.1 renamed DXS2A CAA09804.2, DXS2.2 renamed DXS2B ABI35993.1) ; Chlamydomonas reinhardtii (CAA07554.1) ; *Chrysanthemum x morifolium* (BAE79547.1) ; *Croton stellatopilosus* (BAF75640.1) ; *Deinococcus radiodurans* R1 (NP_295198.1) ; *Elaeis guineensis* (AAS99588.1) ; *Glycine max* (ACO72582.1) ; *Ginkgo biloba* (DXS1 AAS89341.1, DXS2 AAR95699.1) ; *Gossypium barbadense* (ABN13970.1) ; *Hordeum vulgare* (BAJ94232.1. BAJ98888.1) ; *Hevea brasiliensis* (DXS1 clade AAS94123.1, DXS2 clade ABF18929.1; BAF98288.1) ; *Medicago truncatula* (DXS1, CAD22530.1, DXS2, CAN89181.1); *Mentha piperita* (AAC33513) ; *Morinda citrifolia* (AAL32062.1) ; *Narcissus pseudonarcissus* (CAC08458.1) ; *Narcissus tazetta* (ADD82535.1); *Nicotiana tabacum* (CBA12009.1) ; *Oryza sativa* (DXS1 NP_001055524, DXS2 NP_001059086, DXS3 clade BAD67657.1) ; *Ostreococcus lucimarinus* (XP_001416190.1); *Physcomitrella patens* (XP 001771607, XP 001756357, XP 001760423, XP 001754004); *Picea abies* (ABS50519.1 ; ABS50518.1); *Picea sitchensis (ACN39837.1)* ; *Picrorhiza kurrooa LAMIALES (ACB55417.1)* ; *Pinus densiflora* (DXS1 ACC54557.1, DXS2 ACC54554.1) ; *Pinus taeda* (DXS1 ACJ67021.1, DXS2 ACJ67020.1) ; *Populus trichocarpa* (DXS1 XP_002312717.1, DXS2 XP_002303416.1, XP_002331678.1, DXS3 XP_002308644.1) ; *Pueraria montana* (AAQ84169.1) ; *Ricinus communis* (DXS1 XP_002516843.1, DXS2 XP_002533688.1, DXS3 XP_002514364.1 ; DXS4 XP_002532384.1) ; *Salvia miltiorrhiza* (DXS1 ACF21004.1, DXS2 ACQ66107.1) ; *Selaginella moellendorffii* (EFJ14322.1) ; *Sorghum bicolor* (XP_002441088) ; *Solanum lycopersicum* (CAZ66648 ; AF143812_1) ; Solanum tuberosum (DXS1 ADK73609.1) ; *Stevia rebaudiana* (ACI43010) ; Taxus x media (AAS89342.1) ; *Vitis vinifera* (DXS1, XP 002277919 ; DXS2, XP 002266925 ; DXS3, XP 002271585 ; DXS4, XP 002282428 ; DXS5, XP 002270336 ; DXS6, XP 002271782) ; *Zea mays* (DXS1 ABP88134.1, DXS2 : ABP88135.1, DXS3 ADN 22972). Note that *C. roseus* DXS2.1 and DXS2.2 have been renamed DXS2A and DXS2B in this tree.

Enzyme	Primer	Sequence (5’ – 3’)	Amplification
**DXS2.2**	DXS2forA	GGAGATAGTGTATTCTGTGGCCA	3’ end with T7
	DXS2-rev1	CTATCTCCTCCCTTAATTCGTCAGC	5’ end with M13 reverse
	DXS2-full-for	CGACGAGTACTCTAGCATTCCTATCCTC	Full-length ORF
	DXS2-full-rev	CCTAATTCCACAATTGTATTCATCACCAAGTA	
**CMS**	CMS1	GGNCAGCCWATTGCHTTRTA	Internal cDNA fragment
	CMS3	TCATCRGGTGTHGTNACCTT	
	CMS7	AGCGGAGGATGTTGAGAAGGT	3’ end with T7
	CMS5	TCAGGCATGCGGGAGAAAGTA	5’ end with M13 reverse
	CMS11	AGAAGATGTCAATTCTTCGGC	Full-length ORF
	CMS12	GAATTACTATGCAGAGATGGG	
**CMK**	CMKfor	CATGAYYTRGCITCHYTITTTCAT	Internal cDNA fragment
	CMKrev	GTTGCWGCATTRCTRCTYCCWCC	
	3CMKfor1	GGTTCATCTCGATAAAAAGGTGCC	3’ end with T7
	5CMKrev1	GTTATCAGTGCCAGTCTTCTTCCTAT	5’ end with M13 reverse
	CMK-full-for	GGATAGACAAAAAGGCATATCTTGAATG	Full-length ORF
	CMK-full-rev	TGGTTTTGGCATTTATAACCATTTTC	
**HDR**	LytCR8	GAGCGTGCWGTYCARATTG	Internal cDNA fragment
	LytCR10	GAGCGTGCWGTYCARATTG	
	LytCR12	GATCCAGATGAGGATCTCAT	3’ end with T7
	LytCR13	GATTGTGAATAATTTCATTCGTA	5’ end with M13 reverse
	HDR1	ACAATGGCAATCTCTCTCCAA	Full-length ORF
	HDR2	AATCTATGCCAGTTGAAGGGC	

Supplemental Table S1. Details of primer sequences and cloning procedure used to amplify the *DX2.2*, *CMS*, *CMK* and *HDR* cDNAs.

Enzyme	Primer sequence (5’-3’)	Restriction site added	Plasmids *cloning sites*	Complemented *E. coli* strain
**DXS2.2**	**pQE-DXS2-Hindfor** GCAAGCTTGCTGCTGCTCATGGGCATGCT	*Hind*III	pQE-30 *Hind*III	**EcAB4-2 (MG1655 *dxs::CAT MVA*+)**
	**pQE-DXS2-Hindrev** GCAAGCTTCTAAAACTGGAGAACTTGAAGAGTATTCTT	*Hind*III		
**CMS**	**pQE-CMSBam** TCGGATCCGAGAAAAGTGTCTCGGTAATTCT	*Bam*HI	pQE-30 *Bam*HI-*Hind*III	**EcAB4-7 (MG1655 *ygbP::CAT MVA*+)**
	**pQE-CMSHind** TCAAGCTTAGCAGAGATGGGATTCAAAATTCT	*Hind*III		
**CMK**	**pQE-CMKBam** ACGGATCCTGGAATAAGCTAGCTGATGAAGTA	*Bam*HI	pQE-30 *Bam*HI-*Kpn*I	**EcAB4-9 (MG1655 *ychB::CAT MVA*+)**
	**pQE-CMKKpn** TCGGTACCTTCAGAAGACTGGGAGAAGTCT	*Kpn*I		
**HDR**	**pQE-HDR-Bam2** GCGGATCCGAATTCGATGCTAAGAAATTCAGGCAC	*Bam*HI	pQE-30 *Bam*HI-*Hind*III	**MG1655 *ara<>ispH***
	**pQE-HDR-Bam3** GCGGATCCGAACAGAGCACAGAGCTGATGAAC	*Bam*HI		
	**pQE-HDR-Hind** GCAAGCTTCTATGCCAGTTGAAGGGCTTCTTCACG	*Hind*III		

Supplemental Table S2. Details of primer sequences and cloning procedures used to express proteins for functional validation.

Enzyme	Primer	Séquence (5'-3')
**DXS2.1**	qDXS-up	TTGATGTAGGTATTGCCGAGCA
	qDXS-down	ACAATGAGTGGGTCCATCTGC
**DXS2.2**	qDXS2-up	GCTACAGTTGTTGATGCCCGA
	qDXS2-down	TTCAAAGCCAAGAACTGTGCC
**DXR**	qDXR-up	GCAGCAAATGAGAAGGCAGTT
	qDXR-down	GGGCTCAAACCAAGACTGTTC
**CMS**	qCMS-up	GCAACTTTGTCTCCTCAGCATT
	qCMS-down	GCAACTTTGTCTCCTCAGCATT
**CMK**	qCMK-up	AAAGATTGAAGCAGCGTGTGC
	qCMK-down	AGAGGCTTCAGACAAGGAAACG
**MECS**	qMECS-up	TAGAGGATGCGAGGCTCACT
	qMECS-down	TCATAACCTGCCTCGTCCATC
**HDS**	qHDS-up	GTCCCTTACTGAACCTCCAGAG
	qHDS-down	AATCACCTGTCCTGCGTTGG
**HDR**	qHDR-up	GATTGAGAGGACGATGATGCGT
	qHDR-down	AGTTCCACCCTCCAACGACT
**RPS9** AJ749993	qRPS9-for	TTACAAGTCCCTTCGGTGGT
	qRPS9-rev	TTACAAGTCCCTTCGGTGGT

Supplemental Table S3. The sequence of primers used in the qRT-PCR analysis.

Enzyme	Primer sequence (5'-3')	Restriction site added	Plasmids *cloning sites*	Fusion protein
**DXS2.1**	**DXS-GFP-S** ATAGATCTTATGGCGGTTTCCGGGGCT	*Bgl*II	pSCA-cassette-GFPi pSCA-cassette-YFPi *Bgl*II-*Spe*I	DXS-GFP DXS-YFP
	**DXS-GFP-AS** CGACTAGTTTGAAGTTTAAGGGTTCT	*Spe*I		
**DXS2.2**	**DXS2-GFP-S** AGGCTAGCATGGCGGTGGCATCGAAT	*Nhe*I	pSCA-cassette-GFPi pSCA-cassette-YFPi *Spe*I	DXS2-GFP DXS2-YFP
	**DXS2-GFP-AS** CGGCTAGCAAACTGGAGAACTTGAAGAG	*Nhe*I		
**DXR**	**DXR-GFP-S** GGAGATCTTATGGCTTTGAATTCGCTGTCC	*Bgl*II	pSCA-cassette-GFPi pSCA-cassette-YFPi *Bgl*II-*Spe*I	DXR-GFP DXR-YFP
	**DXR-GFP-AS** GGACTAGTGGCAGGGCTCAAACCAAGACT	*Spe*I		
**CMS**	**CMS-GFP-S** CGAGATCTTATGTCAATTCTTCGGCTC	*Bgl*II	pSCA-cassette-GFPi *Bgl*II-*Spe*I	CMS-GFP
	**CMS-GFP-AS** CGACTAGTAGCAGAGATGGGATTCAA	*Spe*I		
**CMK**	**CMK-GFP-S** GCACTAGTATGGCTTCCTCTCAGTCTCTATG TGGT	*Spe*I	pSCA-cassette-GFPi *Spe*I	CMK-GFP
	**CMK-GFP-AS** GCACTAGTCTATTCAGAAGACTGGGAGAAGT CTGT	*Spe*I		
**MECS**	**MECS-GFP-S** TCAGATCTTATGGCTATGGCGACTTCT	*Bgl*II	pSCA-cassette-GFPi *Spe*I	MECS-GFP
	**MECS-GFP-AS** GCACTAGTCTTCCTCATAAGAAGTAC	*Spe*I		
**HDS**	**HDS-GFP-S** GGAGATCTGATGGCGACCGGAACAGTTC	*Bgl*II	pSCA-cassette-GFPi pSCA-cassette-YFPi *Bgl*II-*Spe*I	HDS-GFP HDS-YFP
	**HDS-GFP-AS** GGACTAGTGTCTTCTGCAGGAGGATCAACC	*Spe*I		
**HDR**	**HDR-GFP-S** GCAGATCTTATGGCAATCTCTCTCCAA	*Bgl*II	pSCA-cassette-GFPi *Bgl*II-*Spe*I	HDR-GFP
	**HDR-GFP-AS** TAACTAGTTGCCAGTTGAAGGGCTTC	*Spe*I		

Supplemental Table S4. Details of primer sequences and cloning procedure used to express full-length MEP pathway enzyme fused to FP.

Enzyme	Primer sequence (5’-3’)	Restriction site added	Plasmids *cloning sites*	Fusion protein
**DXS2.1**	**DXS-GFP-S**<br>ATAGATCTTATGGCGGTTTCCGGGGCT	*Bgl*II	pSCA-cassette-GFPi<br>*Bgl*II-*Spe*I	tpDXS-GFP
	**peptideL-DXSrev**<br>GCACTAGTTGCTCTAAGTTCTGCAGCGAGCTG	*Spe*I		
**DXS2.2**	**DXS2-GFP-S**<br>AGGCTAGCATGGCGGTGGCATCGAAT	*Nhe*I	pSCA-cassette-GFPi<br>*Spe*I	tpDXS2-GFP
	**peptideL-DXS2rev**<br>GCGCTAGCGATGGTATCCAAAATAGGAGTAAAAGGCTT	*Nhe*I		
**DXR**	**DXR-GFP-S**<br>GGAGATCTTATGGCTTTGAATTCGCTGTCC	*Bgl*II	pSCA-cassette-GFPi<br>pSCA-cassette-YFPi<br>*Bgl*II-*Spe*I	tpDXR-GFP<br>tpDXR-YFP
	**peptideL-DXRrev**<br>GCACTAGTATTCTCAGCAACTATATCTAGTGTCTG	*Spe*I		
**HDS**	**HDS-GFP-S**<br>GGAGATCTGATGGCGACCGGAACAGTTC	*Bgl*II	pSCA-cassette-GFPi<br>pSCA-cassette-YFPi<br>*Bgl*II-*Spe*I	tpHDS-GFP<br>tpHDS-YFP
	**peptideL-HDSrev**<br>GCACTAGTGTCACTACCAAGGGCTACATTCCC	*Spe*I		

Supplemental Table S5. Details of primer sequences and cloning procedure used to fuse the first 100-residues of DXS2.1, DXS2.2, DXR and HDS to GFP.

Enzyme	Primer sequence (5’-3’)	Restriction site added	Plasmids *cloning sites*	Fusion protein
**DXR**	**DXR-GFP-S**<br>GGAGATCTTATGGCTTTGAATTCGCTGTCC	*Bgl*II	pSCA-cassette-YFPi<br>*Bgl*II-*Spe*I	tpDXR(1-83)-YFP
	**peptideL-DXRrev2**<br>GCACTAGTAACTATTGAAATGGGCTTCTGACCTTC	*Spe*I		
	**DXR-C-for2**<br>GCACTAGTGGCTCTACAGGCTCAGTAGGAACTCAG	*Spe*I	pSCA-cassette-YFPi-tpDXR(1-83)<br>*Nhe*I	Tp(1-83)-YFP-DXR(84-474)
	**DXR-GFP-AS**<br>GGACTAGTGGCAGGGCTCAAACCAAGACT	*Spe*I		
**HDS**	**HDS-GFP-S**<br>GGAGATCTGATGGCGACCGGAACAGTTC	*Bgl*II	pSCA-cassette-YFPi<br>*Bgl*II-*Spe*I	tpHDS(1-100)-YFP
	**peptideL-HDSrev**<br>GCACTAGTGTCACTACCAAGGGCTACATTCCC	*Spe*I		
	**HDS-C-for2**<br>GCACTAGTATGGTTGGGAATGTAGCCCTTGGTAGTGAC	*Spe*I	pSCA-cassette-YFPi-tpHDS(1-100)<br>*Nhe*I	tp(1-100)-YFP-HDS(91-740)
	**HDS-GFP-AS**<br>GGACTAGTGTCTTCTGCAGGAGGATCAACC	*Spe*I		

Supplemental Table S6. Details of primer sequences and cloning procedures used to create internal YFP fusion for DXR and HDS.

Enzyme	Primer sequence (5'-3')	Restriction site added	Plasmids *cloning sites*	Fusion protein
**CAB1**	**CAB1-for** GCACTAGTATGGCCGCCTCAACAATGGC	*Spe*I	pSCA-cassette-CFPi *Spe*I	CAB-CFP
	**CAB1-rev** GCACTAGTCTTTCCGGGAACAAAGTTGGTGGC	*Spe*I		
**TIC40**	**TIC40-for** GCACTAGTATGGAGAACCTTACCCTAGTTTCTTGCT	*Spe*I	pSCA-cassette-CFPi *Spe*I	TIC-CFP
	**TIC40-rev** GCACTAGTACCCGTCATTCCTGGGAAGAGC	*Spe*I		
**CCD4**	**CCD4-for** GCACTAGTATGGACTCTGTTTCTTCTTCTTCCTTCC	*Spe*I	pSCA-cassette-CFPi *Spe*I	CCD4-CFP
	**CCD4-rev** GCACTAGTAAGCTTATTAAGGTCACTTTCCTTGACAAATAAC	*Spe*I		

Supplemental Table S7. Details of primer sequences and cloning procedures used to create plastid subcompartment CFP-markers of. The CAB1 (chlorophyll a/b binding protein 1, At1g29930), TIC40 (translocon at the inner envelope of chloroplasts, At5g16620), CCD4 (carotenoid cleavage dioxygenase 4, At4g19170) proteins of *A. thaliana* were used as a marker of thylakoid, inner membrane of plastid and plastoglobuli, respectively. The coding sequence of these proteins was amplified using retro-transcribed RNA extracted from *A. thaliana* as a matrix.

© 2020 by the authors. Submitted for possible open access publication under the terms and conditions of the Creative Commons Attribution (CC BY) license (http://creativecommons.org/licenses/by/4.0/).
